# Enhancing Mental Wellness in Kenyan Adolescents and Young People: Protocol for a Platform Development and Usability Study

**DOI:** 10.2196/92983

**Published:** 2026-10-15

**Authors:** Paul Macharia, Irene Misoi, Njeri Ngaruiya, Anne Mbwayo, Md Shafiqur Rahman Jabin

**Affiliations:** 1 AHEAD Innovation Hub, Strathmore University, Karen Ole Sangale Rd, off Langata Road, in Madaraka Estate, Nairobi, Nairobi, Nairobi County, Kenya; 2 School of Computing and Engineering Sciences, Stanford University, Palo Alto, CA, United States; 3 Center for Innovations in Global Health, University of Nairobi, Nairobi, Nairobi County, Kenya; 4 Department of Psychiatry, Linnaeus University, Universitetsplatsen 1, Kalmar, 392 31, Sweden, +46 77 228 80 00

**Keywords:** conversational health systems, participatory cocreation, adolescent-centered innovation, psychosocial support tools, stigma-sensitive design, peer-mediated support, scalable digital solutions, low-resource environments, digital health equity, large language models, chatbot

## Abstract

**Background:**

Mental health disorders are a leading cause of disability among adolescents and young people in Kenya, yet access to appropriate and timely support remains severely limited due to shortages of trained professionals, stigma, financial barriers, and geographical inequities. Digital mental health interventions, particularly those leveraging AI, present a promising opportunity to expand access to culturally relevant, low-stigma mental wellness support in low- and middle-income country (LMIC) settings. However, evidence on the feasibility, usability, and acceptability of AI-powered mental health tools for young people in sub-Saharan Africa remains limited.

**Objective:**

This study aims to co-design, develop, and evaluate “Dəˈskəs,” a social media–based digital platform featuring an AI-powered chatbot, to support mental wellness among adolescents and young people in Kenya. The primary objectives are to assess the feasibility of implementation, usability and user experience, acceptability, and the platform’s preliminary applicability across diverse urban, peri-urban, and rural contexts.

**Methods:**

The study uses a human-centered design (HCD) methodology, conducted in 3 phases: formative research and co-design, platform development and iterative refinement, and implementation with monitoring and evaluation. Adolescents and young people aged 18 to 24 years will be purposively recruited from Kibra (urban), Kikuyu Town (peri-urban), and Nachu (rural). Formative data will be collected through focus group discussions to inform platform design, content, and functionality. During implementation, participants will use the platform for 2 months. Quantitative data will include baseline behavioral health screening using the Global Appraisal of Individual Needs–Short Screener (GAIN-SS) and platform use analytics. Qualitative data will be collected through interviews and feedback sessions to assess usability, user experience, acceptability, and perceived value. Data will be analyzed using descriptive statistics and thematic qualitative analysis.

**Results:**

Field activities for the project started in January 2026. Formative data collection was planned to be completed across the 3 Kenyan study sites by the end of February 2026. Usability and user experience testing was planned to run from March 2026 to June 2026. Findings from the formative work, baseline data, and usability and user experience testing will be reported in subsequent original research publications. The study was funded by the Royal Academy of Engineering, UK in 03/2025 and approved by the Strathmore University Institutional Scientific Ethics Review Committee (SU-ISERC; SU-ISERC 2980/25) approved from November 4, 2026.

**Conclusions:**

This protocol describes an adolescent-centered, ethically grounded approach to developing and evaluating an AI-enabled digital mental health intervention in an LMIC context. The findings are expected to contribute to the evidence base on the responsible design and implementation of AI-powered mental health tools for adolescents and young people and to inform future scale-up of accessible, culturally relevant digital mental wellness interventions in Kenya and similar settings.

## Introduction

Mental health conditions represent a significant and growing public health challenge globally, particularly among adolescents [1]. Although mental disorders are recognized as a leading cause of health burden among this age group worldwide, robust data, especially from low- and middle-income countries (LMICs) such as Kenya, remain scarce, often limiting the generalizability of findings. In Kenya, the burden is substantial; data from 2019 indicated that mental disorders were the second leading cause of disability among young people aged 10 to 24 years, contributing significantly to the overall disease burden and accounting for 248,936 disability-adjusted life years (DALYs), representing 9.4% of the total DALYs for this age group [2]. This high burden underscores the profound impact of mental health challenges on young people in Kenya, affecting their education, social development, and future potential, thereby highlighting the need for effective interventions. The economic and social consequences extend beyond impacted individuals, impacting families and national productivity and justifying investment in preventive and early intervention strategies [3,4].

Recent efforts, such as the Kenya National Adolescent Mental Health Survey (K-NAMHS), have begun to provide crucial national prevalence estimates. Findings from a large household survey involving 5155 adolescent-caregiver pairs indicated that 12.1% (95% CI 10.9-13.5) of Kenyan adolescents met the criteria for any mental disorder in the past 12 months, with 9.4% (95% CI 8.3-10.6) meeting criteria in the past 4 weeks. The K-NAMHS report, focusing on children aged 10 to 17 years, similarly found a 12-month prevalence of any mental disorder of 12.2%, with 2.4% experiencing ≥2 disorders. Anxiety disorders emerged as the most common category, affecting 5.6% of adolescents [5].

Specific disorders show sex-related variation within the age group of 10 to 17 years. Attention-deficit/hyperactivity disorder (ADHD) had an overall prevalence of 3.5% but was significantly higher among male individuals (4.7%) than female individuals (2.3%), and among younger adolescents aged 10 to 13 years (4.8%) than those aged 14 to 17 years (2%) [6]. Conduct disorder is estimated to affect 8% overall, with higher rates among male individuals [7]. Conversely, major depressive disorder, with an overall prevalence of 2.1%, was more common in older adolescents (3% vs 1.2%) [5]. These demographic variations suggest that experiences of mental distress manifest differently across development and potentially between sexes, implying that interventions such as “Dəˈskəs,” the study’s custom-made social media–based platform, may benefit from content and engagement strategies tailored to specific age bands, such as early vs late adolescence, and potentially to sex considerations.

Multiple risk factors contribute to this burden in Kenya and the broader sub-Saharan African (SSA) region. These include pervasive socioeconomic challenges such as chronic poverty and unemployment; exposure to violence, conflict, or abuse; conditions such as HIV-AIDS; academic pressure; the negative influences of digital technology and social media; identity formation challenges; and rapid sociocultural changes impacting family structures and values. The recent COVID-19 pandemic has further exacerbated these risks, increasing psychosocial distress for many young people [8].

Despite the evident need, access to and use of mental health services for adolescents in Kenya remain severely limited, reflecting broader challenges across SSA [9]. Systemic barriers are profound, characterized by chronically underfunded public health systems, with mental health often receiving low priority in policy and budget allocation [10]. There is a critical shortage of trained mental health professionals, with estimates for the African region being drastically lower than global averages [11]. This scarcity is often compounded by poor infrastructure and geographical disparities, concentrating limited resources in urban areas.

Social and cultural factors present formidable barriers. Pervasive stigma associated with mental illness leads to discrimination and social isolation and prevents individuals and families from seeking help for fear of negative judgment [12]. Poor mental health literacy, misconceptions about the causes of mental illness, and cultural norms that discourage open discussion of emotional problems further impede help-seeking [13].

At the individual and family level, barriers include a lack of awareness about mental health conditions and available services. Financial constraints, including the affordability of devices and services and the costs of medication and services, are significant obstacles [14,15]. A substantial proportion of caregivers prefer to manage problems within the family, and there can be a lack of trust in formal health care providers or even digital health solutions. Parents act as crucial gatekeepers to care for adolescents, but their own lack of awareness or fear of stigma can prevent access.

Consequently, service use is extremely low. K-NAMHS revealed that only 11.1% of Kenyan adolescents (aged 10-17 years) who were identified as having a mental health problem had accessed any support services in the past year. This aligns with broader estimates suggesting a 90% treatment gap in SSA and indicating that approximately 99% of Africans needing mental health services do not receive them [16]. Strikingly, among the few Kenyan adolescents who did access services, the vast majority turned to informal sources: 34.2% consulted religious or faith leaders, and 31.9% spoke to school staff. Only 10% saw physicians or nurses, and only 2% saw psychiatrists. This pattern underscores a significant disconnect with the formal mental health system, likely driven by accessibility, cost, and stigma concerns [17]. It strongly suggests that effective interventions must either integrate with existing trusted community structures or provide highly accessible, low-stigma alternatives [18,19].

Potential facilitators for improving adolescent mental health care include leveraging settings where adolescents congregate, such as schools, which are recognized as vital entry points. Community-based approaches involving lay health workers or peers can increase reach and reduce stigma [20,21]. Family involvement is also seen as important. Adolescents themselves identify various self-help strategies. These existing coping mechanisms offer valuable insights for developing culturally resonant intervention content [22]. Integrating discussions or activities related to these familiar strategies within “Dəˈskəs” could enhance its relatability and acceptance. Digital technologies offer a promising avenue to overcome many traditional barriers.

## Methods

### Study Design and Population

The research uses a human-centered design (HCD) methodology structured into 3 phases: project initiation and formative research; platform development and content creation; and intervention implementation and monitoring, followed by evaluation and dissemination. The primary study population consists of adolescents and young people in Kenya. For this study, adolescents are defined as participants aged 18 to 19 years, while young people are defined as those aged 20 to 24 years. Recruitment targeted diverse geographical locations, specifically Kibra (urban), Kikuyu Town (peri-urban), and Nachu (rural), to ensure representation across different socioeconomic settings.

A purposive sampling methodology was used to enroll participants from community settings, including sporting events, schools, and youth centers, or through house-to-house outreach conducted by community mobilizers familiar with the local areas. This study was not registered in a public study registry.

### Expertise in the Development Team

The multidisciplinary team is both technically sound and clinically safe. The HCD researcher (led by the principal investigator) is responsible for designing digital health interventions (DHIs) through HCD principles, preparing the research protocol, and leading the usability and user experience (UX) testing of the prototype. This role ensures that the community’s voice leads the technological solution, making the intervention community-led and responsive to the needs of low- and middle-income populations. Complementing this, a clinical psychologist is embedded in the development team to ensure that all chatbot content is developmentally appropriate and clinically sound. Their role involves informing the design and development of the AI intervention, specifically within the context of adolescent mental health. By supporting both the technical and therapeutic aspects, the psychologist ensures that the chatbot’s responses are fine-tuned to achieve desired behavioral outcomes while maintaining clinical integrity.

### The “Dəˈskəs” Intervention and Co-Design Activities

The intervention platform, “Dəˈskəs,’’ derived from the English word “discuss,” is designed to provide a safe online space for connection, peer interaction, and access to reliable health information. Its AI-powered chatbot was iteratively refined through participatory co-design. During the focus group discussions (FGDs), participants identified mental wellness needs and gaps and used sketching and discussion activities to propose chatbot features, navigation elements, language, and content. Feedback from each design cycle was synthesized by the research team and reviewed with the clinical and technical teams. Identified changes were then incorporated into the chatbot’s content, conversational responses, interface elements, or functionality before the subsequent design cycle. This iterative process was conducted through 3 planned co-design cycles, with each cycle informed by feedback from the preceding cycle. Facilitators received standardized orientation to the discussion guide, informed consent procedures, confidentiality, respectful group facilitation, and procedures for responding to distress or safeguarding concerns before conducting the FGDs [23].

The AI-powered chatbot operates using retrieval-augmented generation (RAG) architecture. This architecture separates the retrieval of information from the actual generation of text. When a user submits a query, the text is first processed by Voyage AI’s (MongoDB Inc) embedding model, which translates the user’s natural language into a dense vector, enabling precise semantic searches against a Weaviate vector database that stores the platform’s verified knowledge base for targeted retrieval. The verified knowledge base contains the content listed in Multimedia Appendix 1. The retrieved clinical context, coupled with the user’s recent conversation history, is routed to Claude Sonnet 4 (Anthropic PBC). This approach is intended to ensure that responses are warm, conversational, and strictly grounded in verifiable source documents, minimizing the risk of AI hallucination.

Safety is built directly into the system’s foundation. To maintain a highly controlled environment, the generative model is governed by a strict foundational system prompt designed specifically to counteract prompt injection attacks. If a user attempts to bypass the model’s constraints, alter its designated persona, or reveal its underlying instructions, the system is programmed to ignore the manipulation entirely. Therefore, the chatbot remains locked into its role as a supportive, nonclinical assistant. Supporting these conversational guardrails is a secondary layer of backend infrastructure. The system uses automated token limitations that truncate oversized inputs. This mechanism preempts unexpected behavioral anomalies, guaranteeing that the model operates within safely established processing thresholds at all times.

Beyond managing everyday conversations, the architecture is sensitive to user vulnerability. The language model is explicitly instructed to recognize linguistic markers of severe distress, such as expressions of self-harm or acute crisis. When these critical triggers are detected, the system’s primary objective changes, and it interrupts standard conversational flow to prioritize user safety, rapidly surfacing predefined emergency resources and crisis hotline numbers. Beyond psychological safeguards, the platform also implements strict technical protections. The backend enforces sliding-window rate limiting based on individual client IP addresses. This infrastructure-level constraint (1) mitigates the risk of automated abuse, and (2) ensures that the platform remains highly resilient and accessible to users who need immediate support.

High-risk chatbot interactions, such as those involving self-harm or crisis situations, are designed to trigger 2 actions. First, the chatbot immediately suspends all ongoing conversations. Second, it displays a prompt directing the user to a toll-free phone line where they can speak with a health care provider. This number is available 24 hours a day. The chatbot does not include a human review of conversations or any follow-up support within the app.

### Behavioral Health Screening: The Global Appraisal of Individual Needs–Short Screener

To identify participants’ mental health needs, the study uses the Global Appraisal of Individual Needs–Short Screener (GAIN-SS; Chestnut Health Systems) [24]. This validated tool screens for various psychological and behavioral disorders across 4 primary categories. The internalizing disorder screener (IDScr) focuses on problems such as feeling trapped, lonely, sad, depressed, or hopeless, as well as sleep disturbances and significant anxiety or panic. The externalizing disorder screener (EDScr) screens for behavioral issues, including difficulty paying attention at school or home, and interpersonal conflicts such as bullying or starting fights. The substance disorder screener (SDScr) evaluates the frequency and impact of alcohol or drug use, looking for social problems or withdrawal symptoms. The crime and violence screener (CVScr) identifies issues related to interpersonal violence, such as physical altercations, and illegal activities such as property damage or drug distribution [25-28].

### Data Collection and Analysis

Data collection varied by study objective, using both qualitative and quantitative methods. For the formative research phase, 12 FGDs were conducted across the 3 study locations, with groups stratified by age and sex. Each FGD comprised approximately 6 to 10 participants and lasted approximately 60 to 90 minutes. Discussions followed a semistructured discussion guide covering mental wellness needs, existing sources of support, barriers to help-seeking, preferences for digital support, and desired chatbot functionality. A trained facilitator led each discussion, supported by a member of the research team responsible for field notes and session documentation. With participants’ consent, discussions were audio-recorded and transcribed for qualitative analysis.

In the implementation phase, 40 adolescents per location were planned to be onboarded for 2 months. Quantitative data were planned to undergo univariate and bivariate analysis, while qualitative data from FGDs and interviews were planned to be analyzed using thematic, content, and narrative analysis.

Quantitative data include baseline and follow-up measures and platform use analytics. Engagement metrics include the number and frequency of platform sessions, chatbot interactions, session duration, use of key platform features, and patterns of engagement over the 2-month implementation period. These metrics will be summarized descriptively to characterize uptake, frequency, and intensity of use, and changes in engagement over time. Qualitative feedback will be used to contextualize observed engagement patterns and identify factors influencing continued or discontinued use.

All participant enrollment and follow-up procedures were completed by May 27, 2026. In Nachu, 90 (34.4%) participants were enrolled; in Kibra, 86 (32.8%) were enrolled; and in Kikuyu Town, 86 (32.8%) were enrolled. For the exploratory FGDs, 72 (27.5%) participants were enrolled, 116 (44.3%) were enrolled for the usability testing phase, and 74 (28.2%) completed follow-up for the usability testing. Data analysis is ongoing.

Validated tools, namely the Mobile Application Rating Scale (MARS) [29] and System Usability Scale (SUS) [30], are being used to measure the feasibility, usability, acceptability, and applicability of the app.

### Ethical Considerations

The study was reviewed and approved by the Strathmore University Institutional Scientific Ethics Review Committee (SU-ISERC; SU-ISERC2980/25) and is approved for the period from November 4, 2025, to November 3, 2026. Participation is voluntary, and written informed consent is obtained from all participants before study participation. Participants are informed of the study purpose, procedures, potential risks and benefits, confidentiality protections, and their right to withdraw without penalty. Participants identified with significant mental health concerns are referred to an appropriate public youth-friendly health care facility.

### Data Privacy, Storage, and Access

The platform minimizes the collection of personal information and does not intentionally collect direct identifiers such as names, national identification numbers, or telephone numbers through the chatbot. However, chatbot conversations may contain sensitive mental health information and will therefore be treated as confidential research data [31]. Conversation content retained for research purposes is stored securely and protected using transport layer security (TLS) for data in transit and Advanced Encryption Standard (AES)–256 encryption for data at rest. Technical metadata required for platform operation and evaluation, including time stamps, session information, and use metrics, are limited to the minimum necessary for the study. Access to research data is restricted to authorized members of the research team according to their study roles and responsibilities.

Personal and conversational data will be retained only for as long as necessary to fulfill the approved research purposes and applicable institutional requirements, after which data will be securely deleted, anonymized, or pseudonymized. Conversation data will not be provided to third-party AI providers for their own purposes or used to train external commercial AI models. Any future use of study data for model development or other research purposes will require appropriate ethical and governance review and, where required, additional participant consent.

### Trade-Off Between Anonymity and Longitudinal Monitoring

The decision to minimize the collection of direct identifiers prioritizes privacy, confidentiality, and stigma reduction but limits the ability to link individual chatbot interactions across time, conduct participant-level longitudinal follow-up, and directly contact participants after an interaction. Accordingly, longitudinal assessment will rely primarily on deidentified platform-level use metrics and study assessments collected through the approved research procedures. Safety monitoring will continue through predefined risk triggers, flagged interactions, and human clinical review, with referral procedures activated when significant mental health concerns are identified. The study will therefore prioritize data minimization while retaining information necessary for evaluation and participant safety [32].

## Results

### Overview

The project field activities started in January 2026. Formative data collection was planned to be completed across the 3 Kenyan study sites by the end of February 2026. Usability and UX testing was planned to run from March 2026 to June 2026. Findings from the formative work, baseline data, and usability and UX testing will be reported in subsequent original research publications. The study was funded by the Royal Academy of Engineering, UK in 03/2025 and approved by the Strathmore University Institutional Scientific Ethics Review Committee (SU-ISERC; SU-ISERC 2980/25) approved from November 4, 2026.

### Core Domains

The study is expected to generate several key outcomes across 3 core domains: feasibility of implementation, applicability and clinical impact, and usability and UX of the “Dəˈskəs” platform and its AI-powered chatbot for adolescents and young people in Kenya.

#### Feasibility of Implementation

The research is expected to provide a clear understanding of whether and how the “Dəˈskəs” social media–based platform, including the large language model (LLM)–enabled chatbot, can be implemented within the realities of Kenyan adolescent and youth people’s contexts across urban (Kibra), peri-urban (Kikuyu Town), and rural (Nachu) settings. Findings are expected to identify both enablers and barriers to implementation, including (but not limited to) access to smartphones and connectivity, data affordability, privacy constraints, digital literacy, and contextual preferences that influence uptake and sustained use. The study will also assess the practical feasibility of conducting the 2-month onboarding and monitoring procedures, including recruitment pathways (eg, community events, schools, youth centers, and house-to-house outreach), participant retention, and the operational requirements for safe implementation (eg, escalation or referral processes for participants with significant mental health needs).

#### Applicability and Clinical Impact

The study will explore the applicability and potential mental health impact of “Dəˈskəs” and its chatbot in supporting the mental wellness needs of adolescents and young people. Results are expected to describe how well the platform aligns with users’ lived experiences and expressed priorities identified during formative work, and whether the chatbot and peer-interaction features are perceived as relevant, culturally appropriate, and supportive.

The study will also examine whether engagement with the platform is expected to support earlier recognition of distress, improved access to reliable information, and more timely help-seeking (including referral when appropriate). Baseline screening using the GAIN-SS will be used to characterize participants’ self-reported symptom domains (internalizing, externalizing, substance-related, and crime- or violence-related concerns) and to inform how the intervention can be tailored to the needs of adolescents and young people in these communities. Although the pilot is not designed to establish definitive effectiveness, the findings are expected to provide early evidence of the intervention’s potential to improve mental wellness support in contexts where stigma and service shortages limit access to care.

#### Usability and UX

The study will assess the usability and UX of the “Dəˈskəs” platform and the AI chatbot by examining how adolescents and young people interact with the platform in real-world use over the 2-month testing period. This assessment will evaluate learnability, efficiency, perceived safety, and user satisfaction, including whether the interface, language, tone, and interaction patterns are understandable, acceptable, and engaging.

Results are expected to describe the extent to which users perceive the chatbot as helpful for expressing concerns, seeking guidance, and accessing mental wellness information, as well as how the broader social platform functions (eg, peer interaction and communication features) contribute to acceptability and sustained engagement. Qualitative findings will capture user feedback on relevance, clarity, trust, privacy concerns, and content appropriateness, and will be used to identify specific design and content improvements needed for subsequent iterations of the chatbot and platform.

### Research Impact, Use, and Dissemination of Results

Evaluating the utility of a culturally grounded, human-centered AI chatbot and social platform is crucial for understanding its applicability in addressing mental wellness needs among adolescents and young people in Kenya. Findings are expected to inform strategies for iterative refinement, potential scale-up, and sustained use in LMIC contexts, with attention to equity considerations, including data affordability, device access, and privacy constraints.

The intervention is expected to have a positive impact on mental wellness support processes by improving access to low-stigma support options, strengthening mental health literacy, and enabling more timely pathways to help where needed. Anticipated benefits may include improved user-perceived access to support, increased engagement with reliable information, and enhanced acceptability of digital mental wellness tools among adolescents and young people across diverse settings.

Dissemination of findings will occur at 2 levels. First, preliminary results will be shared with relevant stakeholders through local dissemination forums (eg, stakeholder workshops or conference presentations). Second, findings will be disseminated through peer-reviewed scientific publications and other knowledge translation outputs (eg, policy briefs, youth-friendly summaries, infographics, and social media content), consistent with the protocol’s dissemination plan. Throughout dissemination, the project will adhere to responsible research and publication practices, including ethical conduct, appropriate attribution, and transparency and integrity in reporting.

## Discussion

### Principal Findings

This study is expected to provide evidence on the feasibility, usability, acceptability, and contextual applicability of a human-centered, AI-enabled mental wellness platform for adolescents and young people in Kenya. The anticipated findings are expected to identify contextual factors affecting implementation and engagement, characterize users’ experiences with the chatbot and broader platform, and identify opportunities for iterative refinement. The study is also expected to provide early evidence on whether a culturally grounded digital approach can address barriers to mental wellness support in diverse urban, peri-urban, and rural settings.

### Comparison With Prior Work

Previous research has identified digital mental health interventions as a promising approach for improving access to support among young people, while also highlighting challenges related to engagement, digital access, privacy, and contextual relevance [33]. This study extends that work by combining human-centered co-design with an AI-enabled chatbot and evaluating the intervention across urban, peri-urban, and rural Kenyan settings. The emphasis on contextual adaptation and human oversight is particularly relevant to the responsible implementation of generative AI in health settings [34].

### Reliability and Trustworthiness of AI-Enabled Mental Health Support

Despite the potential of AI-enabled mental health interventions to expand access, LLM-based systems remain vulnerable to inaccurate, misleading, or contextually inappropriate responses, which may undermine user trust and create safety concerns when used for mental health support [34]. The apparent fluency of LLM-generated responses may also lead users to overestimate their reliability or clinical authority [35]. These limitations underscore the importance of bounded use cases, transparent safety constraints, human clinical input, and ongoing monitoring rather than treating the chatbot as a substitute for professional mental health care. The present study will therefore consider not only usability and acceptability but also participants’ perceptions of the relevance, trustworthiness, and safety of chatbot interactions.

### Digital Mental Health Interventions for Young People

DHIs, encompassing mobile apps, web-based platforms, AI, social media, SMS, and wearable sensors, are emerging as a promising approach to addressing mental health needs, particularly for promotion and prevention among young people [36]. The increasing penetration of mobile phones and internet access, even in LMICs, creates potentially scalable and equitable platforms for delivering health support [37].

DHIs hold the potential to circumvent many long-standing barriers to mental health care. They can increase accessibility by overcoming geographical distance and the scarcity of trained professionals. They may reduce travel and consultation costs. Furthermore, the anonymity afforded by some digital platforms can mitigate the pervasive issue of stigma, encouraging help-seeking among those who fear judgment [38]. Social media platforms, in particular, are widely used by adolescents, and some young people prefer using these channels for health communication [39,40].

Several DHIs targeting adolescent mental health are being developed and tested in LMICs and African contexts. These interventions address conditions such as depression, anxiety, and stress and provide support for specific populations, such as adolescents living with HIV. Platforms vary widely, including dedicated apps, web-based programs, interventions delivered via WhatsApp or SMS, and even digital games. Delivery models include self-guided formats, group-based approaches, and interventions supported by lay providers, peers, teachers, or trained professionals [41].

### Challenges and Barriers to Digital Mental Health Implementation

However, significant challenges remain. User engagement and sustained adoption are major hurdles for many DHIs. Practical barriers in resource-limited settings include the high cost of mobile data, limited privacy or access to personal devices, varying levels of digital literacy, and a lack of content available in local languages [42,43]. Concerns about data privacy and confidentiality are also prevalent [44,45]. Critically, the evidence base for the effectiveness of many DHIs in these contexts is still developing, with many studies focusing primarily on feasibility and acceptability rather than clinical outcomes [46].

### Digital Equity and the Need for HCD

The digital divide remains a significant challenge [47,48], and reliance on digital platforms may inadvertently exclude adolescents who have limited or inconsistent access to smartphones, reliable internet connectivity, affordable mobile data, or the digital skills required to use online services effectively [49]. These disparities may be particularly pronounced among young people from socioeconomically disadvantaged households and underserved rural communities. Digital literacy, device sharing, and variability in connectivity may also influence how frequently and independently young people can engage with the platform [50,51]. Therefore, equitable implementation requires attention not only to the availability of the intervention but also to the practical conditions that determine whether young people can access and use it. The inclusion of urban, peri-urban, and rural settings and the use of co-design provide opportunities to identify these barriers and inform adaptations that improve accessibility and usability across different user groups.

### Strengths and Limitations

Key strengths include the use of an HCD approach, engagement of adolescents and young people throughout development, inclusion of diverse geographic settings, and integration of clinical input during intervention development. Limitations include the nonrandomized design, relatively short 2-month implementation period, reliance on self-reported measures, and potential digital-access barriers that may limit participation among young people with limited connectivity or device access.

### Future Directions

Findings from this study will guide refinement of the chatbot and platform and inform subsequent studies assessing effectiveness, longer-term engagement, and scalability. Future research should examine implementation across additional Kenyan settings and assess whether the intervention can be adapted sustainably to other low-resource contexts.

### Conclusions

This study addresses an important gap in evidence on culturally relevant and responsible AI-enabled mental health support for young people in Kenya. By combining youth-informed design with clinical input and evaluation across diverse settings, the study is expected to clarify the practical opportunities and challenges of using AI-enabled digital platforms to support mental wellness. The findings may inform future refinement, implementation, and evaluation of equitable digital mental health interventions in Kenya and other low-resource settings.

## Supplementary material

10.2196/92983 Multimedia Appendix 1 List of validated sources used to develop the AI-powered chatbot knowledge base.

## Abbreviations

ADHD: attention-deficit/hyperactivity disorder
AES: Advanced Encryption Standard
CVScr: crime and violence screener
DALY: disability-adjusted life year
DHI: digital health intervention
EDScr: externalizing disorder screener
FGD: focus group discussion
GAIN-SS: Global Appraisal of Individual Needs–Short Screener
HCD: human-centered design
IDScr: internalizing disorder screener
K-NAMHS: Kenya National Adolescent Mental Health Survey
LLM: large language model
LMIC: low- and middle-income country
MARS: Mobile Application Rating Scale
RAG: retrieval-augmented generation
SDScr: substance disorder screener
SSA: sub-Saharan Africa
SU-ISERC: Strathmore University Institutional Scientific Ethics Review Committee
SUS: System Usability Scale
TLS: transport layer security
UX: user experience

## References

[1] (2021). The state of the world’s children 2021.

[2] Kenya Adolescent Mental Health Group (2023). Burden and risk factors of mental and substance use disorders among adolescents and young adults in Kenya: results from the Global Burden of Disease Study 2019. eClinicalMedicine.

[3] Memiah P, Wagner FA, Kimathi R (2022). Voices from the youth in Kenya addressing mental health gaps and recommendations. Int J Environ Res Public Health.

[4] Wadende P, Sodi T (2023). Mental health literacy: perspectives from Northern Kenya Turkana adolescents. Glob Ment Health (Camb).

[5] Erskine HE, Maravilla JC, Wado YD (2024). Prevalence of adolescent mental disorders in Kenya, Indonesia, and Viet Nam measured by the National Adolescent Mental Health Surveys (NAMHS): a multi-national cross-sectional study. Lancet.

[6] Salari N, Ghasemi H, Abdoli N (2023). The global prevalence of ADHD in children and adolescents: a systematic review and meta-analysis. Ital J Pediatr.

[7] Mohammadi MR, Salmanian M, Keshavarzi Z (2021). The global prevalence of conduct disorder: a systematic review and meta-analysis. Iran J Psychiatry.

[8] Jakobsson CE, Johnson NE, Ochuku B (2024). Meta-analysis: prevalence of youth mental disorders in Sub-Saharan Africa. Glob Ment Health (Camb).

[9] Wakoli D (2024). Exploring the barriers to mental health care and mitigation strategies in Kenya. Int J Res Innov Soc Sci.

[10] Mahomed F (2020). Addressing the problem of severe underinvestment in mental health and well-being from a human rights perspective. Health Hum Rights.

[11] Nicholas A, Joshua O, Elizabeth O (2022). Accessing mental health services in Africa: current state, efforts, challenges and recommendation. Ann Med Surg (Lond).

[12] Subu MA, Wati DF, Netrida N (2021). Types of stigma experienced by patients with mental illness and mental health nurses in Indonesia: a qualitative content analysis. Int J Ment Health Syst.

[13] Sawma T, Hallit S, Younis CJ, Jaber J, Younis C, El Khoury-Malhame M (2024). Cultural stigma, psychological distress and help-seeking: moderating role of self-esteem and self-stigma. medRxiv.

[14] Thomas S, Fylan B, Silcock J, Jabin MS (2025). Medication management using technology-based interventions in older people at home care settings: protocol for a scoping review. JMIR Res Protoc.

[15] Daliri DB, Aninanya GA, Laari TT, Abagye N, Dei-Asamoa R, Afaya A (2024). Exploring the barriers to mental health service utilization in the Bolgatanga Municipality: the perspectives of family caregivers, service providers, and mental health administrators. BMC Health Serv Res.

[16] Saade S, Parent-Lamarche A, Khalaf T, Makke S, Legg A (2023). What barriers could impede access to mental health services for children and adolescents in Africa? A scoping review. BMC Health Serv Res.

[17] Brower KJ (2021). Professional stigma of mental health issues: physicians are both the cause and solution. Acad Med.

[18] Radez J, Reardon T, Creswell C, Lawrence PJ, Evdoka-Burton G, Waite P (2021). Why do children and adolescents (not) seek and access professional help for their mental health problems? A systematic review of quantitative and qualitative studies. Eur Child Adolesc Psychiatry.

[19] Castillo EG, Ijadi-Maghsoodi R, Shadravan S (2019). Community interventions to promote mental health and social equity. Curr Psychiatry Rep.

[20] Hart C, Norris SA (2024). Adolescent mental health in sub-Saharan Africa: crisis? What crisis? Solution? What solution?. Glob Health Action.

[21] Anindhita M, Haniifah M, Putri AM (2024). Community-based psychosocial support interventions to reduce stigma and improve mental health of people with infectious diseases: a scoping review. Infect Dis Poverty.

[22] Fitryasari R, Marthoenis M, Warsini S (2024). Examining the challenges encountered by community health workers and empowering them to address mental health disorders: a qualitative study in Indonesia. Int J Nurs Sci.

[23] Avis JL, van Mierlo T, Fournier R, Ball GD (2015). Lessons learned from using focus groups to refine digital interventions. JMIR Res Protoc.

[24] GAIN instruments. GAIN Coordinating Center.

[25] Carney T, Myers B, Louw J (2016). Reliability of the GAIN-SS, CRAFTT and PESQ screening instruments for substance use among South African adolescents. S Afr J Psychiatr.

[26] Martínez-Loredo V, González-Roz A, Martin-Roig A, Fernández-Artamendi S (2023). New validity evidence of the global appraisal of individual needs-short screener for evaluating internalizing/externalizing behaviors in Spanish adolescents. J Stud Alcohol Drugs.

[27] Dennis ML, Davis JP (2021). Screening for more with less: validation of the Global Appraisal of Individual Needs Quick v3 (GAIN-Q3) screeners. J Subst Abuse Treat.

[28] Khanano R, Barbic S, Henderson J, Mathias S, Richardson CG (2021). Reliability and concurrent validity of the GAIN Short Screener among youth utilizing integrated health services. J Can Acad Child Adolesc Psychiatry.

[29] Molon JN (2025). Mobile App Rating Scale (user version) for the assessment of a community health worker medical application. Mhealth.

[30] Hyzy M, Bond R, Mulvenna M (2022). System usability scale benchmarking for digital health apps: meta-analysis. JMIR Mhealth Uhealth.

[31] (2022). Data Protection Act. Kenya Law.

[32] (2016). Monitoring and evaluating digital health interventions. World Health Organization.

[33] Petrovic M, Cristofolini F, Landoni M, Ionio C, Gaggioli A, Scataglini S, Imbesi S, Marques G (2023). mHealth and Human-Centered Design Towards Enhanced Health, Care, and Well-Being.

[34] (2025). Ethics and governance of artificial intelligence for health: guidance on large multi-modal models. World Health Organization.

[35] Li H, Moon JT, Purkayastha S, Celi LA, Trivedi H, Gichoya JW (2023). Ethics of large language models in medicine and medical research. Lancet Digit Health.

[36] Baka E, Tan YR, Wong BL, Xing Z, Yap P (2025). A scoping review of digital interventions for the promotion of mental health and prevention of mental health conditions for young people. Oxf Open Digit Health.

[37] Marino D, Harutyunyan A, Sargsyan K, Kozlakidis Z, Muradyan A, Sargsyan K (2025). Digitalization of Medicine in Low- and Middle-Income Countries.

[38] Koh J, Tng GY, Hartanto A (2022). Potential and pitfalls of mobile mental health apps in traditional treatment: an umbrella review. J Pers Med.

[39] Ghahramani A, de Courten M, Prokofieva M (2022). "The potential of social media in health promotion beyond creating awareness: an integrative review". BMC Public Health.

[40] Chen J, Wang Y (2021). Social media use for health purposes: systematic review. J Med Internet Res.

[41] Wani C, McCann L, Lennon M, Radu C (2024). Digital mental health interventions for adolescents in low- and middle-income countries: scoping review. J Med Internet Res.

[42] Borges do Nascimento IJ, Abdulazeem H, Vasanthan LT (2023). Barriers and facilitators to utilizing digital health technologies by healthcare professionals. NPJ Digit Med.

[43] Kaboré SS, Ngangue P, Soubeiga D (2022). Barriers and facilitators for the sustainability of digital health interventions in low and middle-income countries: a systematic review. Front Digit Health.

[44] Jabin MS, Mirza A, Ilodibe A, Eldabi T, Vann Yaroson E (2025). Ethical and quality of care-related challenges of digital health twins in care settings for older adults: scoping review. JMIR Aging.

[45] Jabin MS, Yaroson EV, Ilodibe A, Eldabi T (2024). Ethical and quality of care-related challenges of digital health twins in older care settings: protocol for a scoping review. JMIR Res Protoc.

[46] Alhammad N, Alajlani M, Abd-Alrazaq A, Epiphaniou G, Arvanitis T (2024). Patients’ perspectives on the data confidentiality, privacy, and security of mHealth apps: systematic review. J Med Internet Res.

[47] (2025). Digital disparities: tech solutions for Indigenous communities. Open Access Government.

[48] Wepa D, Thomas S, Jabin MS (2025). The experience and impact of digital technologies on Indigenous populations in New Zealand during the COVID-19 pandemic and Cyclone Gabrielle: the Kaupapa Māori methodology. JMIR Aging.

[49] Smith KA, Hardy A, Vinnikova A (2024). Digital mental health for schizophrenia and other severe mental illnesses: an international consensus on current challenges and potential solutions. JMIR Ment Health.

[50] Fitzpatrick PJ (2023). Improving health literacy using the power of digital communications to achieve better health outcomes for patients and practitioners. Front Digit Health.

[51] Koehle H, Kronk C, Lee YJ (2022). Digital health equity: addressing power, usability, and trust to strengthen health systems. Yearb Med Inform.

