# Supplementary material for: Enhancing Mental Wellness in Kenyan Adolescents and Young People: Protocol for a Platform Development and Usability Study
Source: JMIR Res Protoc. 2026 Oct 15;15:e92983. doi: 10.2196/92983 (PMC13671680; doi:10.2196/92983)
Supplement: Supplementary file 1 [file resprot-v15-e92983-s001.docx]

**Appendix 1**

1. World mental health report: transforming mental health for all, ISBN 978-92-4-004933-8 (electronic version), ISBN 978-92-4-004934-5 (print version), World Health Organization 2022
2. NATIONAL GUIDELINES ON WORKPLACE MENTAL WELLNESS Published by: Ministry of Health, Afya House, Cathedral Road, PO Box 30016 Nairobi 00100, <http://www.health.go.ke> 1st Edition 2023
3. Alsamiri YA, Hussain MA, Alsamani OA and Al bulayhi AA (2024) Promoting mental health and wellbeing as means to prevent disability: a review. Front. Public Health 12:1425535. doi: 10.3389/fpubh.2024.1425535
4. Sixth Edition – 2015 Primary Mental Health Service/Gloucestershire Health and Social Care Community have kindly given NHS Somerset permission to adapt this booklet from the original version complied by Alison Sedgwick-Taylor – Clinical Psychologist, Primary Mental Health Service, Gloucestershire NHS Partnership Trust. Original design by: Hobbs Design Ltd. Adaptions to this version made by: Ani Overton Design and Graphics 01258 830 361
5. Child and Adolescent Mental Health: Steps Towards a Policy Framework Name: Prof. Muthoni Mathai, Organisation: University of Nairobi, Department of Psychiatry, Adolescent Mental Summit 2024
6. University of Bristol: The Mental Wellbeing Toolbox Handbook; Lucy Squire, Louisa Slingsby, Sarah Baillie, Updated August 2020
7. MENTAL HEALTH CARE in Settings Where Mental Health Resources Are Limited, An Easy-Reference GUIDEBOOK for Healthcare Providers in Developed and Developing Countries, PAMELA SMITH, MD; ISBN: 978-1-4808-0488-3 (e), ISBN: 978-1-4808-0487-6 (sc), ISBN: 978-1-4808-0489-0 (hc)
8. Mental Health and Physical Activity Toolkit, Guide 1: An introduction to mental health, Mind, 15-19 Broadway, Stratford, London E15 4BQ
9. Wellness Action Plan Guide for people working in a workplace, Mind, 15-19 Broadway, Stratford, London E15 4BQ
10. Personal Wellness Checklist, Health and Wellness Services; University of Colorado Boulder
11. Understanding Your Mental Wellbeing, A Brief Introduction to the Science of Mental Wellbeing, Published by The Wellness Society
12. Youth Mental Wellness Magazine Issue 5: May 2021, CASA, Child and Adolescent Family Mental Health
13. Mental Health Matters, SOCIAL INCLUSION OF YOUTH WITH MENTAL HEALTH CONDITIONS, This publication is based on a background paper prepared by Catherine Bradshaw, Ph.D., M.Ed., University of Virginia and John HopkinsBloomberg School of Public Health, and Amanda Nguyen, Ph.D. Candidate, M.A.; Jeremy C. Kane, M.P.H., Ph.D.; and Judith Bass, Ph.D., M.P.H.: John Hopkins Bloomberg School of Public Health.
14. Guidelines on mental health promotive and preventive interventions for adolescents: helping adolescents thrive, ISBN 978-92-4-001185-4 (electronic version), ISBN 978-92-4-001186-1 (print version), World Health Organization 2020.
15. Wellness Policy Series, Wellness Policy Toolkit: Mental Wellness, June 2024, Global Wellness Institute, 333 S.E. 2nd Avenue, Suite 2048 Miami, FL 33131
16. United Nations System Mental Health and Well-being Strategy for 2024 and beyond
